# Preoperative brain **μ**-opioid receptor availability predicts weight development following bariatric surgery in women

**DOI:** 10.1172/jci.insight.147820

**Published:** 2021-05-24

**Authors:** Henry K. Karlsson, Lauri Tuominen, Semi Helin, Paulina Salminen, Pirjo Nuutila, Lauri Nummenmaa

**Affiliations:** 1 Turku PET Centre, Turku University Hospital and University of Turku, Turku, Finland.; 2 Institute of Mental Health Research, University of Ottawa, Ottawa, Ontario, Canada.; 3 Division of Digestive Surgery and Urology, Turku University Hospital, Turku, Finland.; 4 Department of Surgery, University of Turku, Turku, Finland.; 5 Department of Endocrinology, Turku University Hospital, Turku, Finland.; 6 Department of Psychology, University of Turku, Turku, Finland.

**Keywords:** Endocrinology, Neuroscience, Neuroimaging, Obesity

## Abstract

Bariatric surgery is the most effective method for weight loss in morbid obesity. There is significant individual variability in the weight loss outcomes, yet factors leading to postoperative weight loss or weight regain remain elusive. Alterations in the μ-opioid receptor (MOR) and dopamine D_2_ receptor (D_2_R) systems are associated with obesity and appetite control, and the magnitude of initial brain receptor system perturbation may predict long-term surgical weight loss outcomes. We tested this hypothesis by studying 19 morbidly obese women (mean BMI 40) scheduled to undergo bariatric surgery. We measured their preoperative MOR and D_2_R availabilities using positron emission tomography with [^11^C]carfentanil and [^11^C]raclopride, respectively, and then assessed their weight development association with regional MOR and D_2_R availabilities at 24-month follow-up. MOR availability in the amygdala consistently predicted weight development throughout the follow-up period, but no associations were found for D_2_R. This is the first study to our knowledge to demonstrate that neuroreceptor markers prior to bariatric surgery are associated with postoperative weight development. Postoperative weight regain may derive from dysfunction in the opioid system, and weight loss outcomes after bariatric surgery may be partially predicted based on preoperative brain receptor availability, opening up new potential for treatment possibilities.

## Introduction

The prevalence of obesity is constantly increasing and reaching global pandemic levels. Accumulating evidence suggests that dysfunctions in appetite control and reward processing mechanisms significantly contribute to weight gain and maintenance, and particularly, the brain’s dopamine and opioid systems in the reward circuit are dysfunctional in obesity. Dopamine D_2_ receptor (D_2_R) expression and function are altered in obesity (1–3), whereas the endogenous opioid system is consistently linked to hedonic aspects of feeding in animals (4, 5). In humans, feeding triggers endogenous opioid release (6), and accordingly, pharmacological challenge studies have found that both μ-opioid receptor (MOR) antagonists and inverse agonists reduce human eating behavior (7, 8). MOR levels are also downregulated in obese subjects, underlining the importance of opioid system perturbation in overeating (9, 10).

Bariatric surgery is currently the most effective method for weight loss in obesity. Mean postoperative total weight loss of 27% has been shown among patients even after 12 years (11). Bariatric surgery procedures are also much more effective than intensive medical therapy to reach glycemic control (12). For weight loss, there is currently some consensus to use standardized reporting guidelines in bariatric surgery literature (13), but similar uniform consensus needs to be achieved regarding postoperative weight regain in order to assess the durability of weight loss and to reliably evaluate potential treatment options (14). Weight regain following bariatric surgery occurs in one-fifth (15–17) up to one-third of the patients (18–20).

Factors leading to weight regain following surgery remain poorly understood, yet cross-sectional studies point toward a possible role of the brain in regulating the treatment response. Impulsivity and disinhibition are traits often associated with poorer weight loss after surgery, but both psychosocial issues and psychiatric comorbidities may also have a major impact on weight loss outcomes (21–24). However, only few neuroimaging studies have examined neural predictors of weight loss after surgery. To our knowledge, there are only 2 small MRI studies that have investigated brain markers that might affect the weight loss outcome of the surgery. Functional connectivity and alterations in brain activity in some of the key areas of reward circuit predict weight loss 12 months after sleeve gastrectomy (25, 26). However, the role of specific neurotransmitter systems — such as D_2_R and MOR implicated in feeding and reward processing — in postsurgical weight gain and loss remain unknown. In the present study, we addressed this issue by measuring obese subjects’ MOR and D_2_R availability with positron emission tomography (PET) before they underwent bariatric surgery. We followed the subjects for 2 years and predicted their weight loss outcomes with regional MOR and D_2_R availabilities. We show that MOR availability particularly in the amygdala predicts long-term outcome of bariatric surgery, suggesting a causal role of this region in appetite control and food intake.

## Results

**Figure 1:**
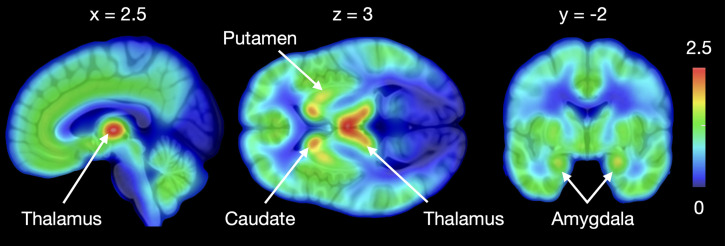
Mean [^11^C]carfentanil BP_ND_ in morbidly obese subjects before surgery. *BP*_ND_, ratio of specific to nondisplaceable binding in the brain.

**Figure 2:**
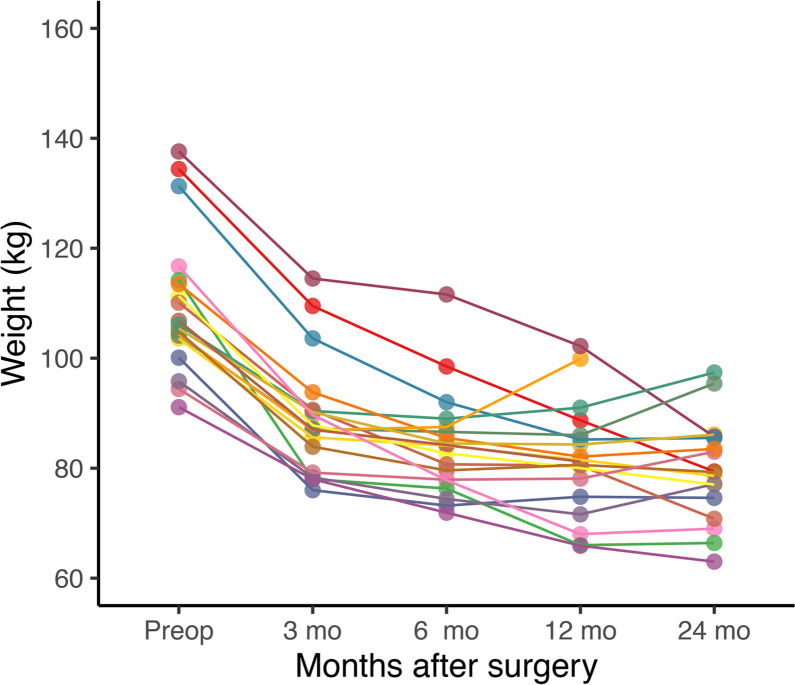
Weight development after bariatric surgery for each subject (*n* = 19). Two subjects discontinued the study before the 24-month follow-up visit.

Mean MOR availability in the subjects is presented in Figure 1. As reported earlier (9, 27), preoperative BMI was negatively correlated with MOR availability in all the tested regions (mean *r* = –0.56). Mean weight loss at 3 months was 20.8 ± 5.6 kg, at 6 months was 25.7 ± 7.7 kg, at 12 months was 28.3 ± 12.1 kg, and at 24 months was 30.7 ± 15.1 kg. Postoperative weight development is shown in Figure 2. Roux-en-Y gastric bypass was performed on 6 subjects and sleeve gastrectomy on 13 subjects. The effects of different surgery types on MOR availability and weight loss were not analyzed because of insufficient statistical power.

**Figure 3:**
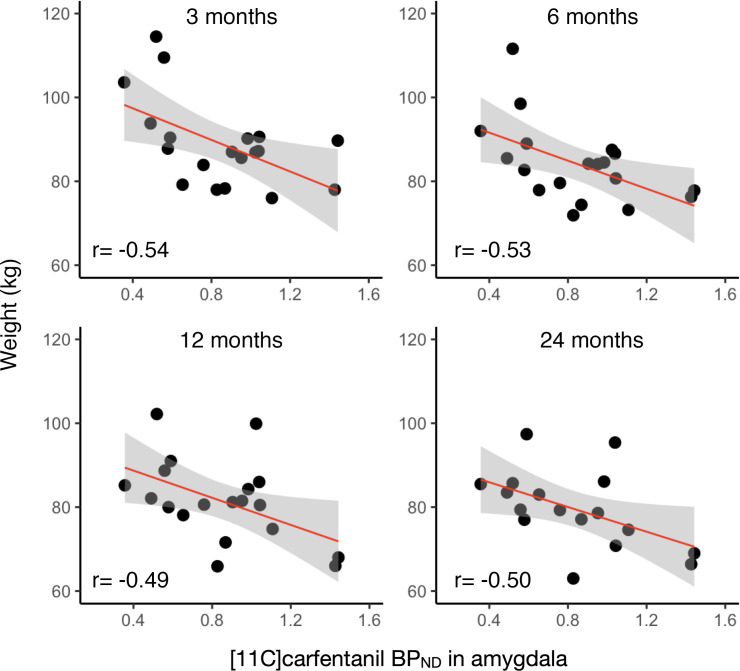
Correlations between preoperative [^11^C]carfentanil *BP*_ND_ in the amygdala and subject weight at 3, 6, 12, and 24 months.

Correlations between preoperative MOR availabilities and subject weight are shown in Table 1. Preoperative MOR availabilities were significantly associated with the subject weight in the amygdala (*r* = –0.54) (Figure 3), insula (*r* = –0.46), ventral striatum (*r* = –0.48), and putamen (*r* = –0.49) at 3 months. A significant association was also found in the amygdala at 6 months (*r* = –0.53) and at 12 months (*r* = –0.49) (Figure 3). Moreover, significant association was observed in the amygdala (*r* = –0.50) (Figure 3) and thalamus at 24 months (*r* = –0.49).

Preoperative weight did not correlate with MOR availabilities in any brain area. We did not find any significant correlation between preoperative D_2_R availability and subject weight in any brain area at any time point. No significant correlations between Beck Depression Inventory II (BDI-II) and State-Trait Anxiety Inventory (STAI) scores and MOR and D_2_R availabilities in any brain area were observed. BDI-II and STAI scores did not predict weight loss at any time point.

Five subjects experienced clinically substantial weight regain (median 6.4 kg). We could not find a significant association between weight gain and receptor availabilities.

## Discussion

Our main finding was that neuroimaging markers predicted weight development after bariatric surgery. MOR availability in the amygdala consistently predicted weight development throughout the 24-month follow-up, even though MOR availability was not initially associated with preoperative weight. MOR availabilities were predictive of future gross weight but not with weight change normally evaluated using standardized outcome definitions of percentage excess weight loss (%EWL), percentage excess BMI loss (%EBMIL), or percentage total weight loss (%TWL). No associations were found for D_2_R. These results show that neuromolecular phenotypes may contribute to the outcome of weight loss after bariatric surgery, possibly providing novel predictive biomarkers for postoperative weight loss after bariatric surgery. However, our finding suggestive of a potential predictive impact of MOR availability in postoperative weight loss needs to be evaluated in larger patient cohorts.

Obesity is expensive for society, especially because of the obesity-related comorbidities. Bariatric surgery reduces mean costs to the health service compared with usual care (28). However, a significant number of the patients experience weight regain (18), which was also seen in our study (Figure 2). Determining patient characteristics leading to sustainable weight loss long term is important, but so far there have not been reliable markers. Some metabolic markers may predict weight regain after surgery (29); also taste preference for salty or sucrose-sweetened foods may contribute to some extent (30, 31). Our study is the first PET study to our knowledge to predict the outcome of bariatric surgery from neuroimaging markers, and only 2 small MRI studies exist (25, 26). Smith et al. also showed that Roux-en-Y gastric bypass can lead to increased weight loss in subjects who have a preference for sweet foods, which was also coupled with specific changes in ventral tegmental area response assessed by functional MRI (30). Our study shows that molecular organization of the brain’s reward circuit is an important determinant of surgery-induced weight loss.

Bariatric surgery alleviates depressive and anxious symptoms (32, 33), yet psychiatric comorbidities are associated with weight gain following surgery (23, 24). Surgery has a more positive impact on the depressive disorders than anxiety disorders (34), but preoperative symptoms also likely affect the results of the surgical methods. Preoperative depression is also associated with lower postoperative weight loss (35). Although MOR availability is associated with depressive and anxious symptoms (36), we observed no association between depressive and anxiety symptoms and weight loss. This may be due to low statistical power for the questionnaire-based measures, as well as relative crudeness of questionnaires (in comparison with structured interviews, such as the Montgomery-Asberg Depression Rating Scale).

Human PET studies have shown that feeding activates the endogenous opioid system (6), and consequently, dysfunction of the endogenous opioid system is a key component underlying overeating and thus a feasible target for pharmacological and behavioral interventions. Previous studies have investigated effects of bariatric surgery and following weight loss on separate receptor systems, showing mainly unaltered D_2_R availability and normalized MOR availability (27, 37–39), although 2 animal studies have yielded contradictory findings (40, 41). Our study highlights the importance of MOR in the amygdala in predicting weight management after the surgery. Opioidergic circuits in the amygdala are critical for emotions including fear and anxiety (42), but it is also one of the key regions in appetite control (43). MOR availability in the amygdala is associated with subclinical depressive and anxiety symptoms (36), and individual differences in MOR availability in the amygdala may explain individual differences in eating behavior (44). It has also been shown that bariatric surgery can recover initially downregulated MOR in the amygdala of obese patients (27).

Our study has several limitations. Only female subjects were studied, and the results may not be generalizable to male subjects. Sample size was relatively small, possibly precluding establishing associations between MOR availabilities, weight development, and preoperative psychiatric symptoms. However, the original power analysis suggested that the study has sufficient power (27), and the employed radioligand has high affinity for MOR (45) and high test-retest reliability (46), further improving the validity of the data. We followed the subjects for only 2 years as part of their standard clinical visits, but longer follow-up might have shown different trajectories. However, longer follow-up studies are planned (47).

In summary, preoperative MOR availability in the amygdala predicts weight outcomes after bariatric surgery. Postoperative weight regain or primary weight loss failure may partially depend on a dysfunctional opioid system. There is growing evidence that the opioidergic system plays an important role in governing a multitude of reward functions (44), and this study confirmed its significance in the aspects of feeding (6). Downregulation of the MOR system can be reversed by surgical (27) and nonsurgical weight loss (10). The present study extended these findings by establishing the role of MOR in long-term weight maintenance. Future prospective studies should address whether MOR availability is also predictive of weight gain in normal-weight subjects and whether it predicts weight loss success by conventional dieting-based approaches.

## Methods

### Study population.

We studied 19 morbidly obese women (mean BMI 40, mean age 43) scheduled to undergo bariatric surgery, i.e., Roux-en-Y gastric bypass or sleeve gastrectomy, according to their standard clinical treatment. Subjects were recruited from Turku University Hospital outpatient care and they were White Caucasians. Subject characteristics are shown in Table 2. Clinical screening of the subjects included history, physical examination, anthropometric measurements, and laboratory tests. Exclusion criteria for this study involved opiate drug use, neurological and severe mental disorders, substance abuse, and excessive alcohol consumption determined by clinical interviews, medical history, and blood tests. Seven subjects were smokers (3–15 cigarettes per day). Antidiabetic, antihypertensive, and cholesterol-lowering drugs were paused prior to the study. Subject weight was recorded before surgery as well as at 3, 6, 12, and 24 months after surgery during standard hospital visits. Two subjects dropped out of the study before the 24-month follow-up visit, but their weight data at 3, 6, and 12 months were included in the analysis. Baseline depressive and anxiety symptoms were recorded using BDI-II and STAI, respectively (48, 49).

### Image acquisition and quantification of receptor availability.

We measured MOR availability with the high-affinity agonist [^11^C]carfentanil (45) and D_2_R availability with the antagonist [^11^C]raclopride (50) using PET. Brain scans were performed before the start of the standard very low-calorie diet. Radiotracer production has been described previously (9). [^11^C]carfentanil and [^11^C]raclopride scans were performed on separate days. Both radiotracers had high radiochemical purity (>99%). Before scanning, a catheter was placed in the subject’s left antecubital vein for tracer administration. The head was strapped to the scanner table in order to prevent head movement. Subjects fasted 2 hours prior to scanning. A CT scan was performed to serve as an attenuation map. Clinical well-being of subjects was monitored during the scanning.

We injected both tracers as bolus (252.2 ± 10.8 MBq [^11^C]carfentanil and 248.4 ± 21.9 MBq [^11^C]raclopride). After the injection, radioactivity in the brain was measured with the GE Healthcare Discovery 690 PET/CT scanner (General Electric Medical Systems) for 51 minutes, using 13 time frames. MRI was performed with Philips Gyroscan Intera 1.5T CV Nova Dual scanner to exclude structural abnormalities and to provide anatomical reference images for the PET scans. High-resolution anatomical images (1 mm^3^ voxel size) were acquired using a T1-weighted sequence (repetition time 25 ms, echo time 4.6 ms, flip angle 30°, scan time 376 seconds).

All alignment and coregistration steps were performed using SPM8 software (www.fil.ion.ucl.ac.uk/spm/) running on MATLAB R2012a (The MathWorks Inc.). To correct for head motion, dynamic PET images were first realigned frame to frame. The individual T1-weighted MRI images were coregistered to the summation images calculated from the realigned frames. Regions of interest (ROIs) for reference regions were drawn manually on MRI images using PMOD 3.4 software (PMOD Technologies Ltd.). The occipital cortex was used as the reference region for [^11^C]carfentanil and the cerebellum for [^11^C]raclopride. Receptor availability was expressed in terms of *BP*_ND_, which is the ratio of specific to nondisplaceable binding in the brain. *BP*_ND_ was calculated applying the basis function method for each voxel using the simplified reference tissue model with reference tissue time activity curves as input data (51).

### Statistics.

The subject-wise parametric *BP*_ND_ images were normalized to the Montreal Neurological Institute space using the T1-weighted MRI images and smoothed with a Gaussian kernel of 8 mm full width half maximum. Anatomic ROIs were generated in ventral striatum, dorsal caudate, putamen, insula, amygdala, thalamus, orbitofrontal cortex, anterior cingulate cortex, medial cingulate cortex, and posterior cingulate cortex using the AAL (52) and Anatomy (53) toolboxes. Regional [^11^C]carfentanil and [^11^C]raclopride binding potentials (*BP*_ND_) were extracted and correlated with subject weights at 3, 6, 12, and 24 months after surgery. Moreover, BDI and STAI scores were correlated with [^11^C]carfentanil and [^11^C]raclopride binding potentials as well as subject weight at different time points. A *P* value less than 0.05 was considered significant. Group differences in receptor availabilities between normal-weight and morbidly obese subjects have been previously reported for a subset of the subjects (9, 27).

### Study approval.

The study was conducted in accordance with the Declaration of Helsinki and approved by the Ethics Committee of the Hospital District of Southwest Finland, Turku, Finland (SleevePET2, NCT01373892, http://www.clinicaltrials.gov). All participants signed ethics committee–approved informed consent forms prior to scans.

## Author contributions

LN and PN designed the experiments. PS recruited the study subjects. SH produced the radiotracers. HKK acquired PET data. HKK and LT analyzed PET data. HKK, LT, SH, PS, PN, and LN wrote the manuscript, interpreted the data, and submitted the manuscript.

## Figures and Tables

**Table 1:**
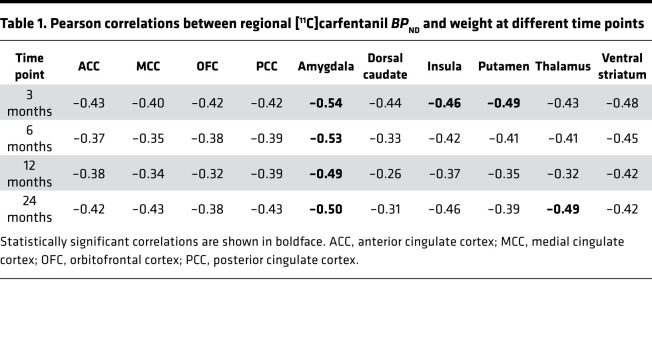
Pearson correlations between regional [^11^C]carfentanil *BP*_ND_ and weight at different time points

**Table 2:**
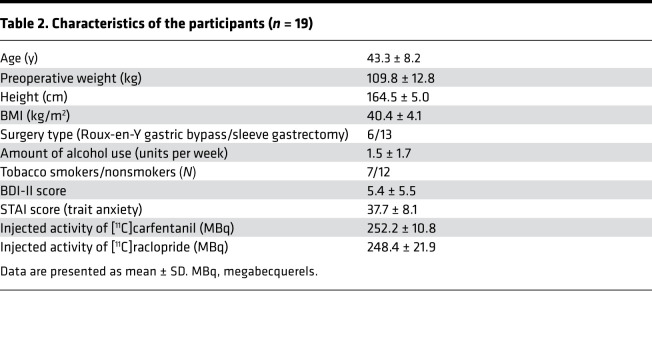
Characteristics of the participants (*n* = 19)

## Version 1 04/13/2021

In-Press Preview

## Version 2 05/24/2021

Electronic publication
